# Three Decades of Food and Drug Administration Authorizations of Artificial Intelligence/Machine Learning-Enabled Medical Devices: Persistent Specialty Concentration and the Care-Delivery Gap (1995–2025)

**DOI:** 10.7759/cureus.112583

**Published:** 2026-07-13

**Authors:** Pouyan Golshani, Mary S Joseph

**Affiliations:** 1 Interventional Radiology, Kaiser Permanente Los Angeles Medical Center, Los Angeles, USA; 2 Radiology, University of California, Riverside, Riverside, USA

**Keywords:** ai in cardiology, artificial intelligence (ai), clinical decision support, healthcare policy, health informatics system, radiology ai, virtual care delivery

## Abstract

Background

The US Food and Drug Administration (FDA) maintains a public list of artificial intelligence (AI) and machine learning (ML)-enabled medical devices that have received marketing authorization. Prior published analyses examined this list at earlier time points and reported a marked dominance of radiology applications.

Methodology

We performed a longitudinal descriptive analysis of all 1,430 AI/ML-enabled medical device authorizations recorded by the FDA between September 1995 and December 2025 to characterize the cumulative growth, specialty distribution, and manufacturer concentration of authorized devices.

Results

The annual authorization volume increased from a mean of 1.8 per year between 1995 and 2014 to 264 per year between 2023 and 2025, with 331 authorizations recorded in 2025 alone. Devices reviewed by the FDA’s Radiology panel accounted for 1,094 of 1,430 authorizations (76.5%), and the three most represented panels (Radiology, Cardiovascular, and Neurology) accounted for 90.6% of all authorizations. Several large clinical specialties were represented by very small numbers of authorized devices, including Pathology (n = 9, 0.6%), Microbiology (n = 6, 0.4%), and Obstetrics and Gynecology (n = 4, 0.3%). No authorizations were recorded under a psychiatry or behavioral health review panel. Of 740 unique companies, 502 (67.8%) had a single authorized device, while 13 (1.8%) companies accounted for 247 (17.3%) devices.

Conclusions

The cumulative regulatory record demonstrates rapid growth that has been concentrated in image-rich diagnostic specialties, with limited representation across many specialties that account for substantial clinical activity in the United States. These findings may inform policy discussions about where regulatory, infrastructure, and dataset investments are most needed to broaden the clinical scope of medical AI.

## Introduction

The number of artificial intelligence (AI) and machine learning (ML)-enabled medical devices that have received marketing authorization from the US Food and Drug Administration (FDA) has grown rapidly over the past decade [1]. The FDA maintains a public list of these devices, which it updates periodically and which has been the basis of multiple published analyses characterizing the regulatory landscape [1-3]. An early review identified 64 AI/ML-based medical devices through 2020 [1]. A subsequent landscape analysis using the same FDA list identified 691 devices by October 2023 [2]. A more recent taxonomic review examined 1,016 authorizations to characterize the underlying AI tasks performed by these devices [3]. Across these analyses, devices reviewed by the FDA’s Radiology panel have consistently represented the majority of authorizations [1-3].

Despite the cumulative growth of the regulatory record, several gaps in published analyses remain. First, to our knowledge, no published analysis has characterized the full cumulative dataset through the end of calendar year 2025, a period during which authorization volume continued to accelerate. Second, prior analyses have generally reported the radiology share without quantifying the corresponding underrepresentation of other clinical specialties relative to their burden of clinical activity. Third, the concentration of authorizations among a small number of repeat-authorizing manufacturers has not been systematically described, despite implications for market structure and innovation incentives.

In this report, we describe the cumulative regulatory record of AI/ML-enabled medical devices authorized by the FDA between September 1995 and December 2025. We characterize the growth trajectory by year, the distribution of authorizations across the FDA’s lead review panels, the temporal stability of specialty concentration, and the distribution of authorizations across manufacturers.

A preprint version of this manuscript was previously posted to the medRxiv preprint server on May 8, 2026 (DOI: https://doi.org/10.64898/2026.05.08.26352766).

## Materials and methods

Data source

This analysis used the FDA’s publicly maintained list of AI/ML-enabled medical devices, accessed in a deidentified tabular form [4]. The FDA-maintained list reports devices that have received marketing authorization through the 510(k), De Novo, or Premarket Approval (PMA) pathways and that the FDA has identified as containing AI/ML functionality [4]. The dataset contained 1,430 authorizations with final decision dates between September 29, 1995, and December 30, 2025. The complete authorization dataset analyzed in this study, together with the analysis scripts used to reproduce all figures and statistics, has been deposited in a public GitHub repository (https://github.com/GigHz-com/fda-aiml-devices-dataset) and archived on Zenodo with a citable DOI (https://doi.org/10.5281/zenodo.20336151); analyses reported here used the FDA’s source list directly.

Variables

For each authorization, the dataset contained the date of final decision, submission number, device name, manufacturer (company), the FDA lead review panel (an FDA classification corresponding broadly to the medical specialty of intended use), and the primary product code. Submission numbers were used to classify authorizations by regulatory pathway: numbers beginning with “K” were classified as 510(k); numbers beginning with “DEN” as De Novo; and numbers beginning with “P” as PMA.

Analysis

Descriptive analyses were performed using Python 3.11.7 with the pandas (v2.2.0) and matplotlib (v3.8.x) libraries. The FDA list was retrieved on December 31, 2025, and the analyzed snapshot is archived at Zenodo (DOI: 10.5281/zenodo.20336152, v1.0.0) for reproducibility. The FDA publishes each authorization record (510(k) clearance, De Novo grant, or PMA approval) as a separate row; the analysis treats each row as one authorization event and does not deduplicate supplemental clearances of the same device. No additional data cleaning or imputation was performed. We computed annual and cumulative authorization counts by year of final decision, the proportion of authorizations attributable to each FDA lead review panel, and the distribution of authorized devices across unique manufacturers. To examine the temporal stability of specialty concentration, we computed the proportion of authorizations attributable to the Radiology panel and to the top three combined panels for each year between 2015 and 2025. The earlier period (1995-2014) was excluded from year-stratified specialty analysis because of small annual counts. No formal hypothesis tests were planned; analyses were descriptive.

Ethics

This analysis used a publicly available regulatory dataset that contained no patient-level information. The applicability of institutional review board oversight was considered and determined to be inapplicable.

## Results

Cumulative growth, 1995-2025

**Figure 1:**
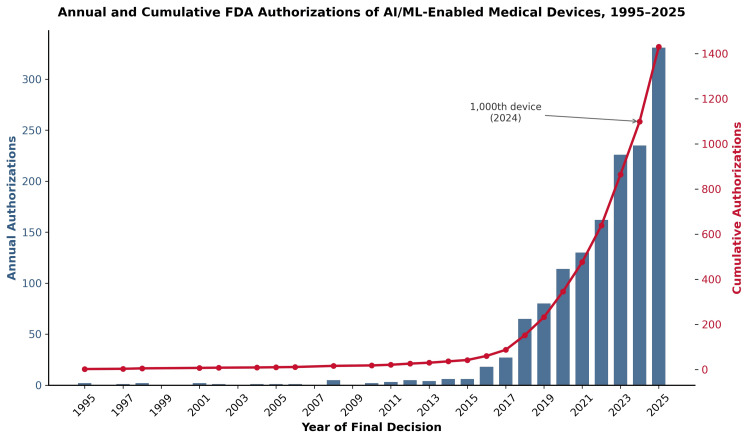
Annual and cumulative FDA authorizations of AI/ML-enabled medical devices, 1995–2025. FDA = Food and Drug Administration; AI = artificial intelligence; ML = machine learning

A total of 1,430 AI/ML-enabled medical device authorizations were recorded between September 29, 1995, and December 30, 2025. The mean annual authorization rate was 1.8 per year between 1995 and 2014, increasing to 39.2 per year between 2015 and 2019, 135.3 per year between 2020 and 2022, and 264.0 per year between 2023 and 2025. The annual authorization count reached a single-year maximum of 331 authorizations in 2025, representing 23.1% of the all-time cumulative total. The cumulative count crossed 100 authorizations (7.0% of the all-time total) in 2018, 500 (35.0%) in 2022, and 1,000 (69.9%) in 2024 (Figure 1).

Specialty distribution

**Figure 2:**
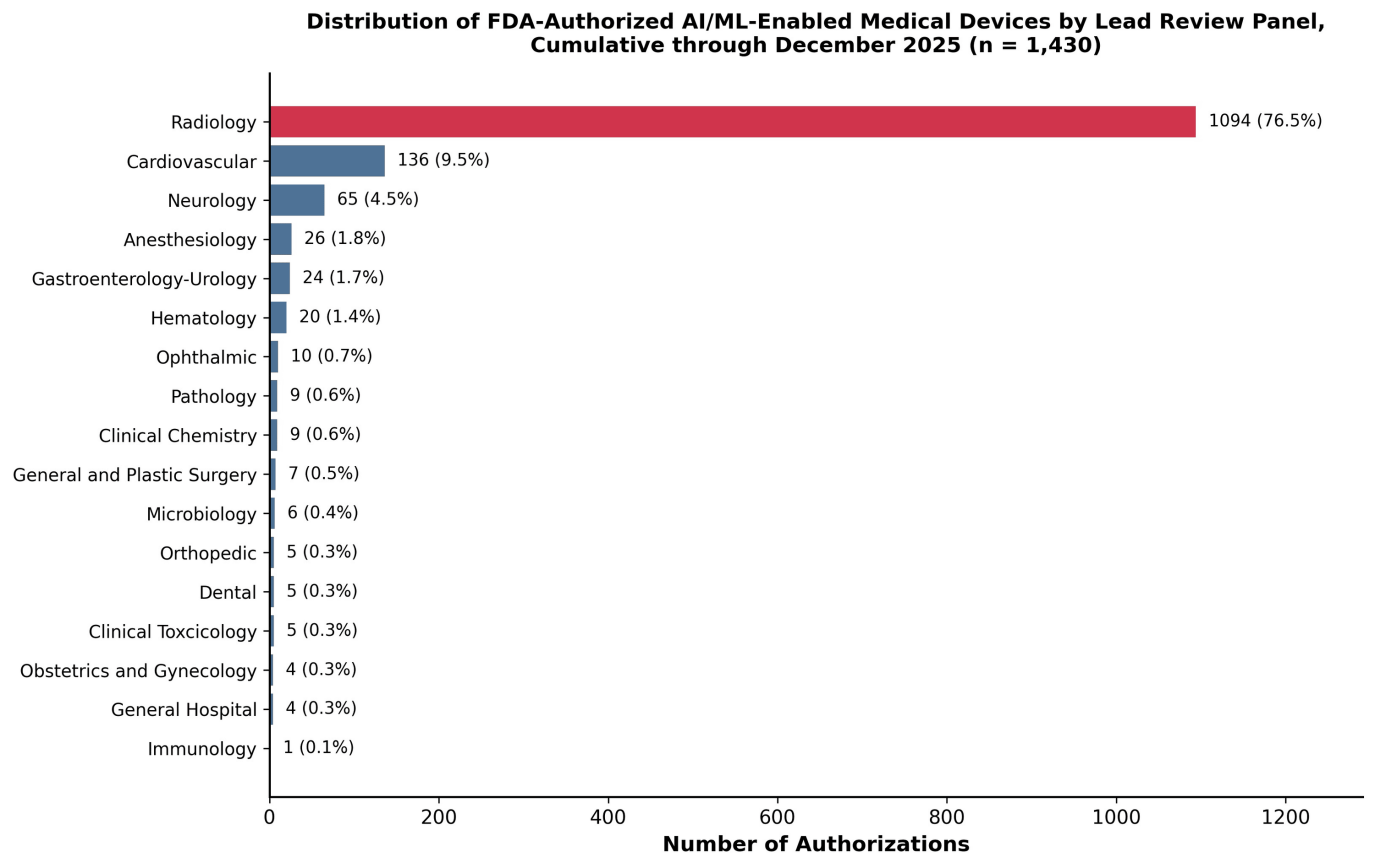
Cumulative distribution of FDA-authorized AI/ML-enabled medical devices by lead review panel, 1995–2025 (n = 1,430). FDA = Food and Drug Administration; AI = artificial intelligence; ML = machine learning

Authorizations were unevenly distributed across the FDA’s lead review panels. The Radiology panel accounted for 1,094 of 1,430 cumulative authorizations (76.5%), the Cardiovascular panel for 136 (9.5%), and the Neurology panel for 65 (4.5%). The three most represented panels collectively accounted for 1,295 (90.6%) authorizations, and the five most represented panels accounted for 1,345 (94.1%) authorizations (Figure 2).

Several panels corresponding to large clinical specialties were associated with small absolute numbers of authorizations. The Pathology panel was associated with nine (0.6%) authorizations, the Microbiology panel with six (0.4%) authorizations, the Dental panel with five (0.3%) authorizations, and the Obstetrics and Gynecology panel with four (0.3%) authorizations. No authorizations in the dataset were classified under a psychiatry or behavioral health review panel.

**Figure 3:**
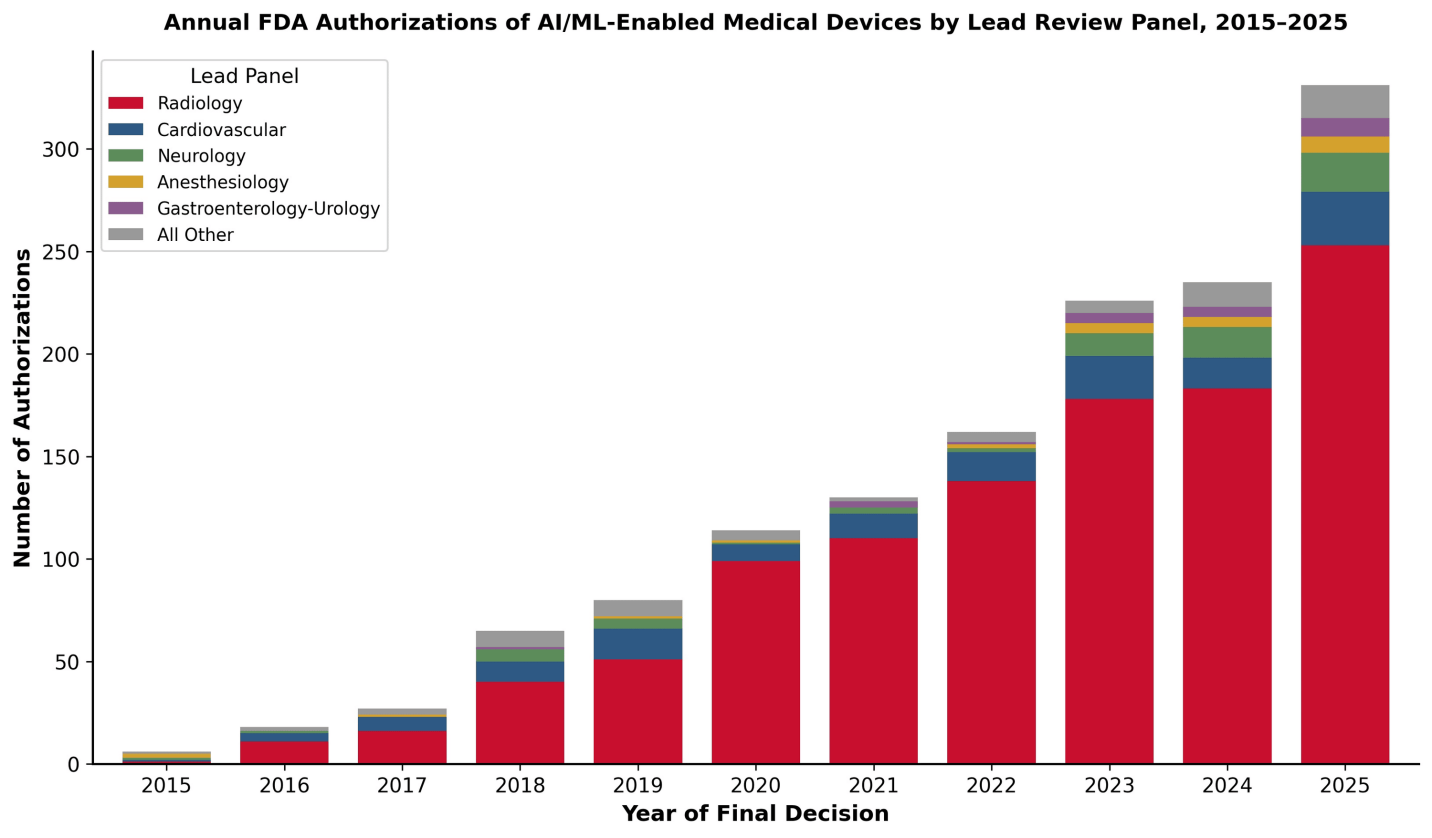
Annual FDA authorizations of AI/ML-enabled medical devices by lead review panel, 2015–2025. FDA = Food and Drug Administration; AI = artificial intelligence; ML = machine learning

The radiology share of annual authorizations was stable over time. Between 2018 and 2025, the proportion of annual authorizations reviewed by the Radiology panel remained between 61.5% and 86.8%, with no sustained downward trend across the most recent decade (Figure 3). In each of the years 2020 through 2025, the Radiology panel accounted for at least 76% of new authorizations.

Regulatory pathway

Of 1,430 authorizations, 1,376 (96.2%) were cleared through the 510(k) pathway, 37 (2.6%) through the De Novo pathway, and 17 (1.2%) through the PMA pathway. The 510(k) share is consistent with prior reports identifying 510(k) clearance as the predominant authorization mechanism for AI/ML-enabled devices, including a recent analysis of the 2024 cohort that reported 94.6% of that year’s AI/ML authorizations cleared via 510(k) [2,3,5].

Manufacturer concentration

**Figure 4:**
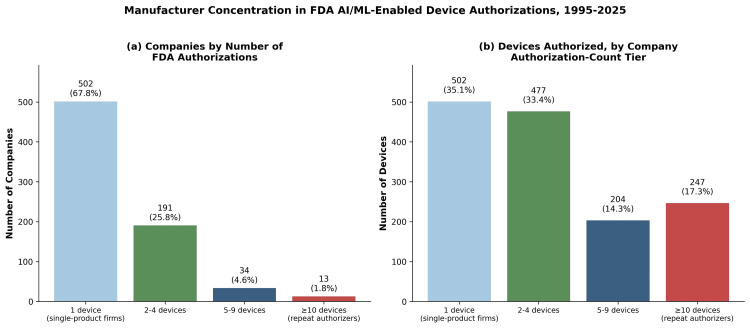
Manufacturer concentration in FDA AI/ML-enabled device authorizations, 1995–2025. (a) Distribution of the 740 unique manufacturers by the number of FDA AI/ML device authorizations they hold, binned into four tiers (1, 2–4, 5–9, and ≥10 authorizations). The majority (502 of 740, 67.8%) are single-product firms, while only 13 (1.8%) firms hold ≥10 authorizations. (b) Total number of authorized devices (n = 1,430) attributable to firms in each of the same four tiers. The 13 repeat-authorizing firms (≥10 authorizations each) collectively account for 247 of 1,430 devices (17.3%), illustrating the disproportionate market share held by a small group of repeat manufacturers despite single-product firms outnumbering them roughly 39:1. FDA = Food and Drug Administration; AI = artificial intelligence; ML = machine learning

The 1,430 authorizations were associated with 740 unique manufacturers. Of these, 502 (67.8%) manufacturers had a single authorization in the dataset. A total of 13 (1.8%) manufacturers had 10 or more authorizations and collectively accounted for 247 of 1,430 authorizations (17.3%). The 10 manufacturers with the highest authorization counts accounted for 217 (15.2%) authorizations, and the 25 manufacturers with the highest counts accounted for 336 (23.5%) authorizations (Figure 4). The single manufacturer with the highest authorization count had 51 authorizations.

## Discussion

We performed a longitudinal descriptive analysis of all 1,430 AI/ML-enabled medical device authorizations recorded by the FDA between September 1995 and December 2025. Our analysis extends prior published reports of this regulatory record [1-3] by approximately two additional years and approximately 414 additional authorizations relative to the most recent published taxonomic analysis [3]. Three findings warrant discussion: the persistent concentration of authorizations within a small number of imaging-related specialties, the very small number of authorizations corresponding to several specialties with substantial clinical volume in the United States, and the bimodal distribution of authorizations across manufacturers.

The persistent dominance of radiology in the FDA AI/ML authorization record has been described in earlier analyses [1-3]. Our updated cumulative figure of 76.5% for the radiology share is consistent with reports from earlier time points. The stability of this share over a decade, despite a roughly 50-fold increase in the annual authorization rate, suggests that the underlying drivers are structural rather than transitional [6]. Plausible structural drivers include the relative availability of large, labeled, standardized imaging datasets through Digital Imaging and Communications in Medicine (DICOM) infrastructure, the universal standard format and exchange protocol for medical imaging that enables interoperable storage and transfer of images across vendors and institutions, and that has historically facilitated assembly of training datasets at scale in radiology that are not as readily available in other specialties; the comparatively well-defined regulatory framework for image analysis software [7]; and the existence of established commercial pathways for incorporating image analysis software into radiology workflows.

The corresponding underrepresentation of other clinical specialties is the more consequential finding. Several specialties that account for substantial volumes of clinical activity in the United States were associated with very small numbers of authorizations in the cumulative record, a pattern noted in earlier commentary on the uneven trajectory of medical AI across clinical domains [8]. The absence of authorizations under a psychiatry or behavioral health review panel is particularly notable given the substantial population burden of mental illness and the active research literature on AI applications in mental health, where digital and algorithmic tools have advanced but have not translated into FDA-authorized devices at a meaningful rate [9]. Similarly, the small number of authorizations under the Pathology, Microbiology, and Obstetrics and Gynecology panels stands in contrast to the breadth of clinical activity in those specialties.

The manufacturer-level findings indicate a market structure in which a small number of repeat-authorizing firms account for a disproportionate share of authorizations, while most firms in the dataset have a single authorization. This pattern is consistent with both an emerging industry interpretation, in which many small firms compete in early stages, and a smaller number consolidate over time, and with a barriers to entry interpretation, in which the resources required for repeat regulatory engagement, including infrastructure, validation, and post-market surveillance capabilities, widely recognized as obstacles to clinical AI translation [10], favor larger, established firms.

Limitations

This analysis has several limitations. First, the FDA’s published list of AI/ML-enabled devices is curated by the agency, and prior work has noted that the list is updated irregularly and does not include every device that may incorporate AI/ML functionality [3,11]. Second, the FDA’s lead review panel classification reflects regulatory categorization and may not align exactly with clinical specialty-level care delivery. Third, the dataset analyzed here did not contain information on indication for use, target patient population, performance characteristics, or post-market experience [12]. Fourth, the analysis was descriptive and did not include formal hypothesis testing. Fifth, the FDA’s lead review panel reflects regulatory categorization and may not capture cross-specialty applications; behavioral-health or psychiatric AI tools, for example, may have been authorized under non-psychiatric panels (e.g., sleep-related devices under Anesthesiology), and the absence of authorizations under a dedicated psychiatry/behavioral health panel therefore represents the absence of that specific regulatory classification rather than necessarily the absence of all behavioral health AI authorizations. Finally, our manufacturer concentration analysis treated each company name as a unique entity and did not account for corporate parent-subsidiary relationships, mergers, or acquisitions.

## Conclusions

FDA authorizations of AI/ML-enabled medical devices through 2025 remain concentrated within a small set of imaging-adjacent specialties, with limited representation across most clinical disciplines and no authorizations recorded under a dedicated psychiatry or behavioral health review panel. The descriptive pattern is consistent with structural advantages in specialties that have long-established digital workflow infrastructure, mature commercial pathways, and large labeled datasets, although our analysis does not test causation. The accompanying market structure, dominated by single-authorization firms alongside a small number of repeat authorizers, is consistent with both an emerging industry interpretation and a barriers to entry interpretation. We do not interpret these findings as evidence of market failure or as a basis for prescribing any specific policy response. Whether and how the observed specialty concentration evolves will depend on a combination of market, infrastructure, dataset, and regulatory factors that future work could examine in greater depth.
